# Supplementary material for: The 3D structure of lipidic fibrils of α-synuclein
Source: Nat Commun. 2022 Nov 10;13:6810. doi: 10.1038/s41467-022-34552-7 (PMC9649780; doi:10.1038/s41467-022-34552-7)
Supplement: Supplementary file 1 — Supplementary Information [file 41467_2022_34552_MOESM1_ESM.pdf]

Supplementary Information

The 3D structure of lipidic fibrils of α-synuclein

Benedikt Frieg[1*], Leif Antonschmidt[2*], Christian Dienemann[3], James A. Geraets[1], Eszter E. Najbauer[2], Dirk Matthes[4], Bert L. de Groot[4], Loren B. Andreas[2], Stefan Becker[2], Christian Griesinger[2,5], and Gunnar F. Schröder[1,6]

[1] Institute of Biological Information Processing (IBI-7: Structural Biochemistry) and JuStruct: Jülich Center for Structural Biology, Forschungszentrum Jülich; Jülich, Germany.

[2] Department of NMR-Based Structural Biology, Max Planck Institute for Multidisciplinary Sciences; Göttingen, Germany.

[3] Department of Molecular Biology, Max Planck Institute for Multidisciplinary Sciences; Göttingen, Germany.

[4] Department of Theoretical and Computational Biophysics, Max Planck Institute for Multidisciplinary Sciences; Göttingen, Germany.

[5] Cluster of Excellence "Multiscale Bioimaging: From Molecular Machines to Networks of Excitable Cells" (MBExC), University of Göttingen; Göttingen, Germany.

[6] Physics Department, Heinrich Heine University Düsseldorf; Düsseldorf, Germany.

*These authors contributed equally to this work.

Correspondence and requests for materials should be addressed to Gunnar F. Schröder (gu.schroeder@fz-juelich.de) and Christian Griesinger (cigr@mpinat.mpg.de).

Supplementary figures and legends

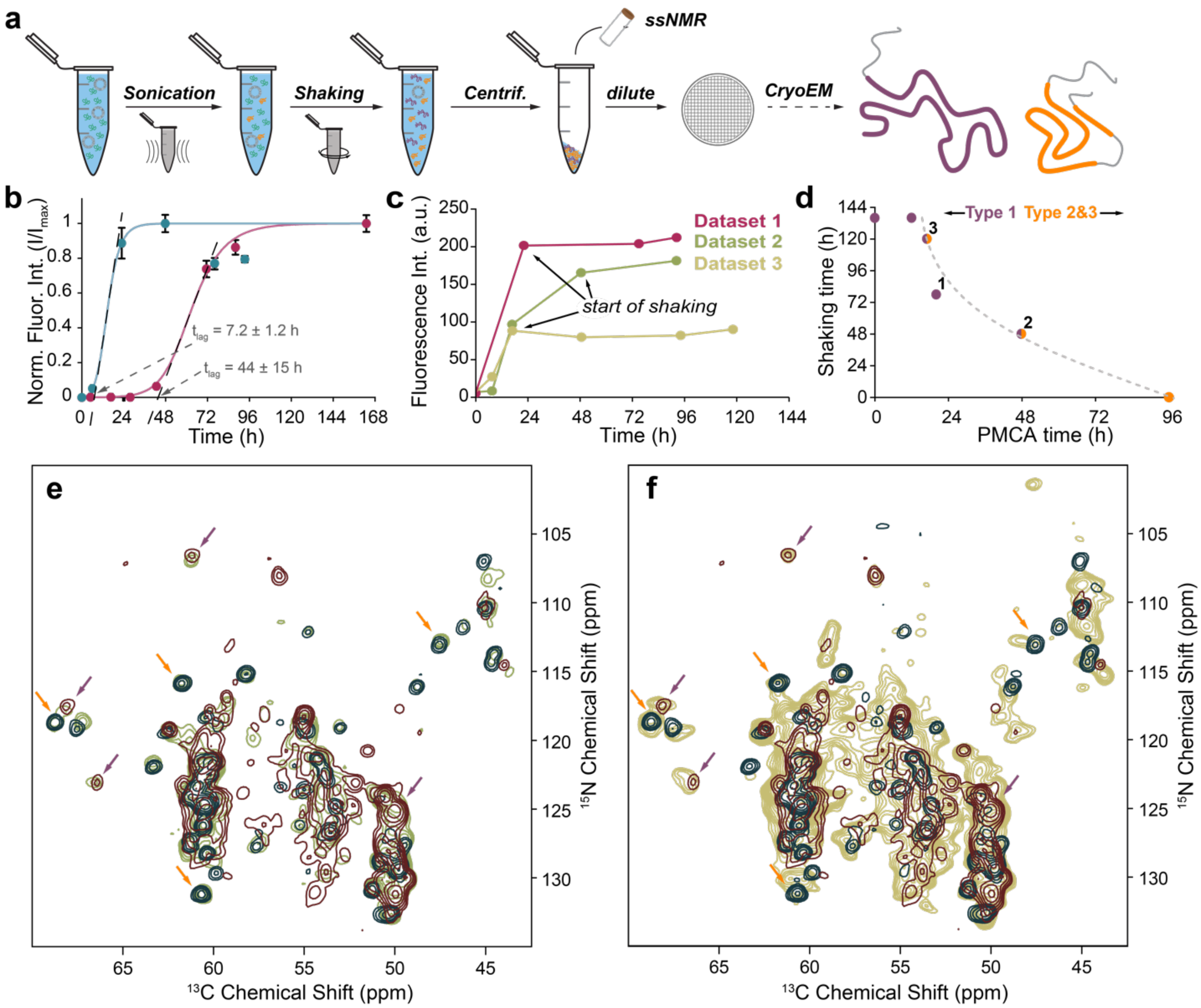

Supplementary Figure 1 | Preparation and characterization of fibril samples.
a, Workflow schematic for preparation of αSyn fibrils in this study. **b,** Representative curves of normalized ThT fluorescence (I/I_{max}) following the aggregation kinetics of αSyn in the presence (blue) and absence (magenta) of vesicles of POPA and POPC (1:1) under PMCA conditions. Curves were obtained by fitting the data to an unseeded secondary nucleation model using Amylofit[1] (www.amylofit.ch.cam.ac.uk/). Data are presented as mean values +/- SD averaged over two scans. Lag-times t_{lag} were determined as the intersection of the x-axis and a linear function fitted to the steepest part of the curve **c,** ThT fluorescence data of individual samples analyzed by cryo-EM. **d,** Correlation plot of times spent under different agitation conditions. Points are color coded by the dominant fibril types. Characterization of type L1 (purple) and type L2 & L3 (orange) was done by ssNMR (fibril subtypes were indistinguishable) and in labelled cases by Cryo-EM (datasets 1-3). **e,** (H)CANH spectra of αSyn fibrils used for dataset 1 acquired at 800 MHz with 55 kHz MAS (green) and **f,** (H)NCA of αS fibrils used for dataset 2 acquired at 850 MHz with 17 kHz MAS (yellow) compared to spectra of fibrils prepared under purely PMCA (blue, 950 MHz, 100 kHz MAS) and shaking conditions (red, 1200 MHz, 55 kHz MAS). Arrows indicate characteristic peaks originating from either L1 (purple) or L2/L3 fibrils (orange), showing that in

either sample a mixture of both fibril types is present. Spectra of fibrils prepared under PMCA conditions (blue) are reproduced from ref.[2].

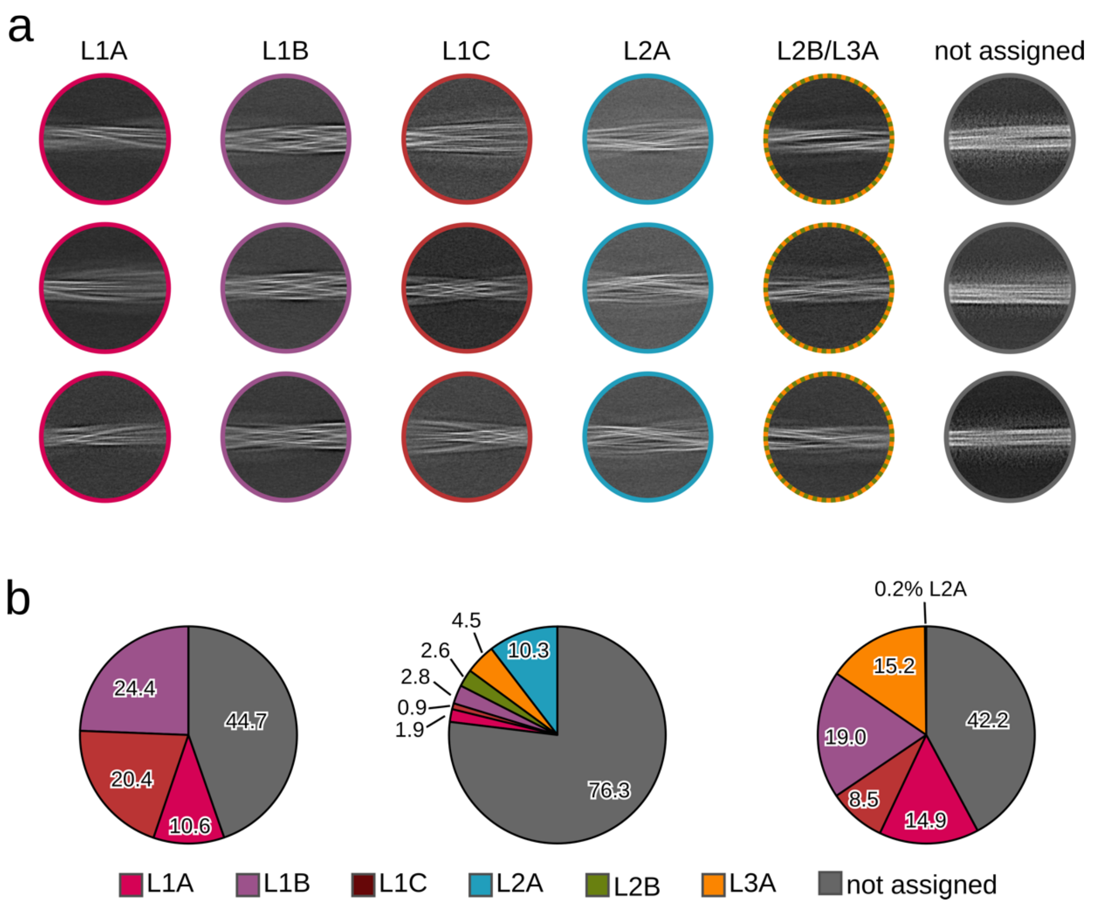

Supplementary Figure 2 | Distribution of lipid-induced αSyn fibrils.
a, Representative 2D class averages for all lipid-induced αSyn fibrils and segments that could not be assigned to any of the polymorphs after 2D classification, due to the lack of well-defined and clear filament features. Instead, the unassigned classes are not sharp and partially very fuzzy at the fibril surface. **b**, Pie charts visualizing the relative population (labels in %) of each lipid-induced αSyn fibril polymorphs in dataset 01 (left) 02, (middle), and 03 (right).

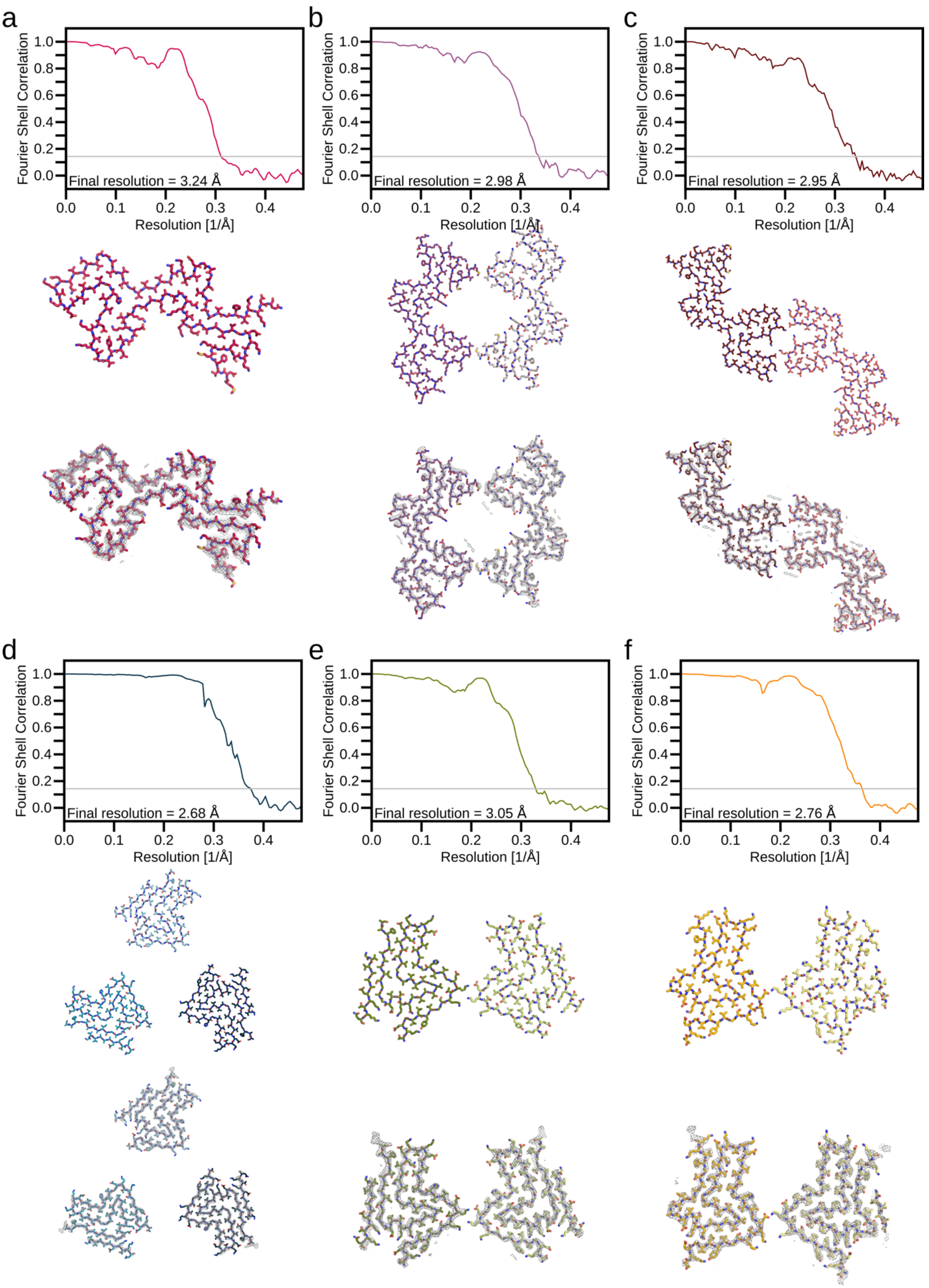
a
b
c
d
e
f
Fourier Shell Correlation
Resolution [1/Å]
Final resolution = 3.24 Å
Final resolution = 2.98 Å
Final resolution = 2.95 Å
Final resolution = 2.68 Å
Final resolution = 3.05 Å
Final resolution = 2.76 Å
1.0
0.8
0.6
0.4
0.2
0.0
0.1
0.2
0.3
0.4

Supplementary Figure 3 | Fourier shell correlation curves, final cryo-EM density maps, and atomic model of the lipidic αSyn fibrils.
Masked-corrected (z-percentage is 0.1) Fourier shell correlation (FSC) curves (top panels), the atomic model (middle panels), and a superposition of the atomic model and the central slice of the density map with a width of 10.5 Å (10 pixel, 1.05 Å/pixel; gray isomesh) (lower panels) for L1A (**a**), L1B (**b**), L1C (**c**), L2A (**d**), L2B (**e**), and L3A (**f**). The final resolution is shown in the plot and was estimated from the value of the FSC curve for two separately refined masked half-maps at 0.143 (gray line).

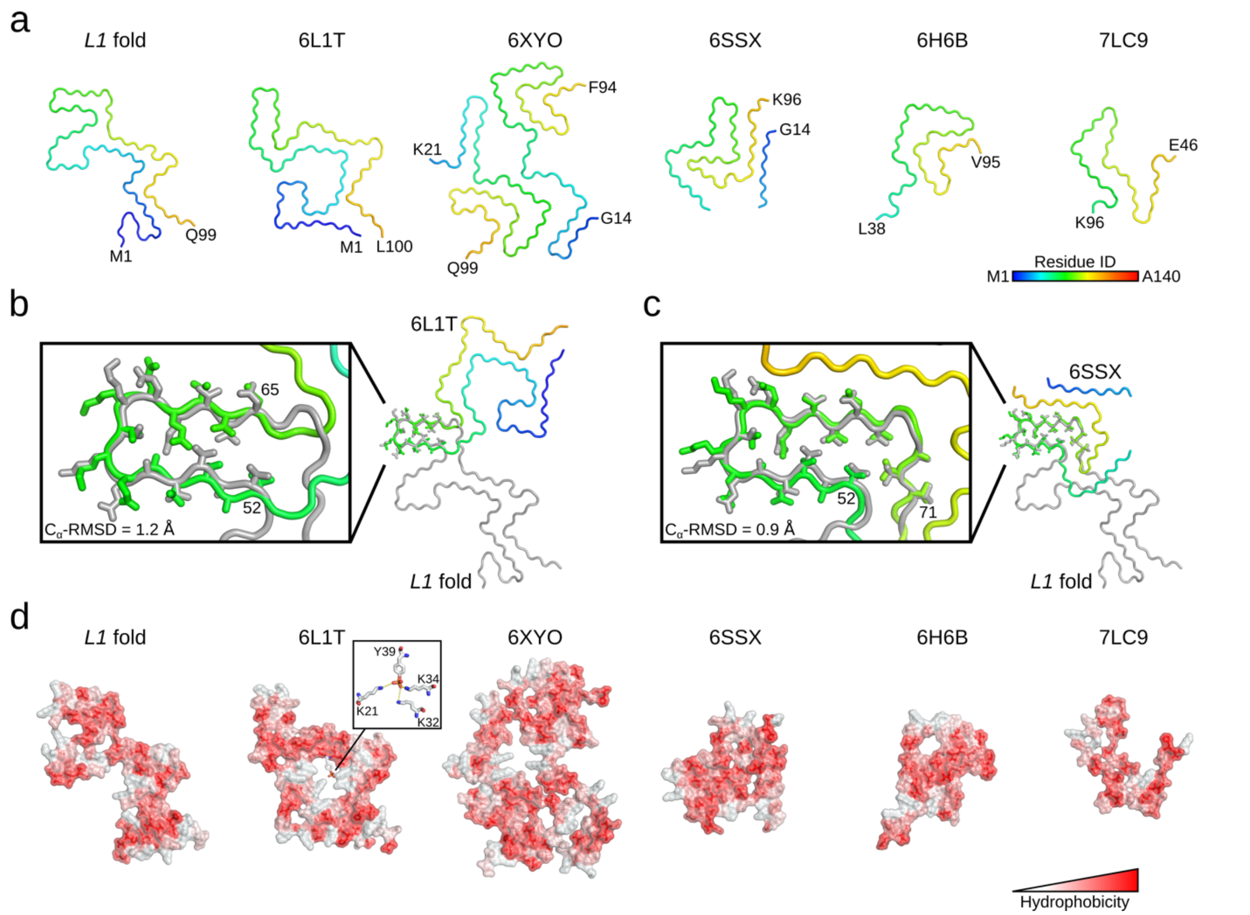

Supplementary Figure 4 | Comparison of the lipidic *L1* fold and known structures.
a: Backbone traces of previously resolved αSyn structures, with residues colored according to the rainbow pallet in the lower right corner. The four-letter PDB-ID is reported with the structures (6L1T: protofilament fold of Y39 phosphorylated αSyn[3]; 6XYO: fold of MSA Type I filaments[4]; 6SSX: protofilament fold of polymorph 2[5]; 6H6B: protofilament fold of polymorph 1[6]; 7LC9: protofilament fold of the structure of the N-terminal αSyn truncation 41-140[7]). The labels depict the N- and C-terminal residues. **b**, **c**: Superposition of the lipidic L1 fold (gray) with PDB-ID 6L1T (**b**) and PDB-ID 6SSX (**c**). The residues considered for the superposition are shown as sticks in the close-up view, along with the Cα-RMSD of the superposition. **d**: Stick-surface models from (**a**), but now colored according to the Eisenberg hydrophobicity scale[8]. For PDB-ID 6L1T, the close-up view shows interactions mediated by the phosphorylated Y39, which form the core of the protofilament fold.

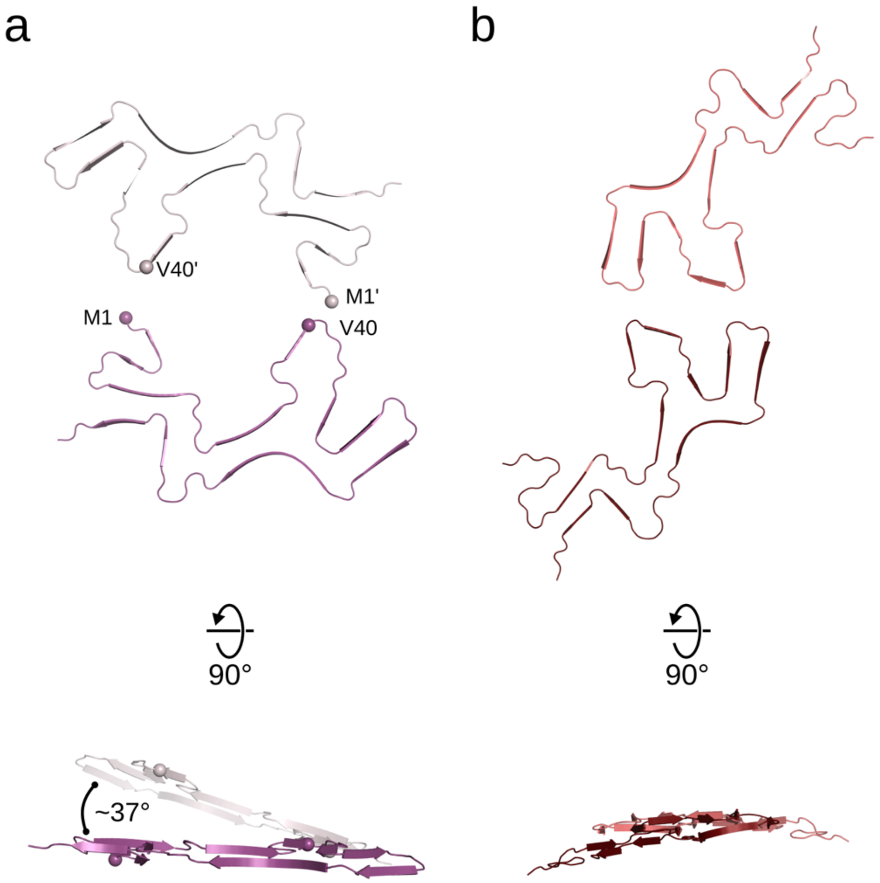

Supplementary Figure 5 | The tilting of protofilaments in the *L1B* fibril.
Two central protein chains extracted from the L1B (**a**) and L1C (**b**) αSyn fibril models in top and side-view. To estimate the tilt angle between the protofilaments in L1B, we measured the dihedral angle described by the Cα atoms of M1-V40-M1’-V40’ of two opposite protein chains.

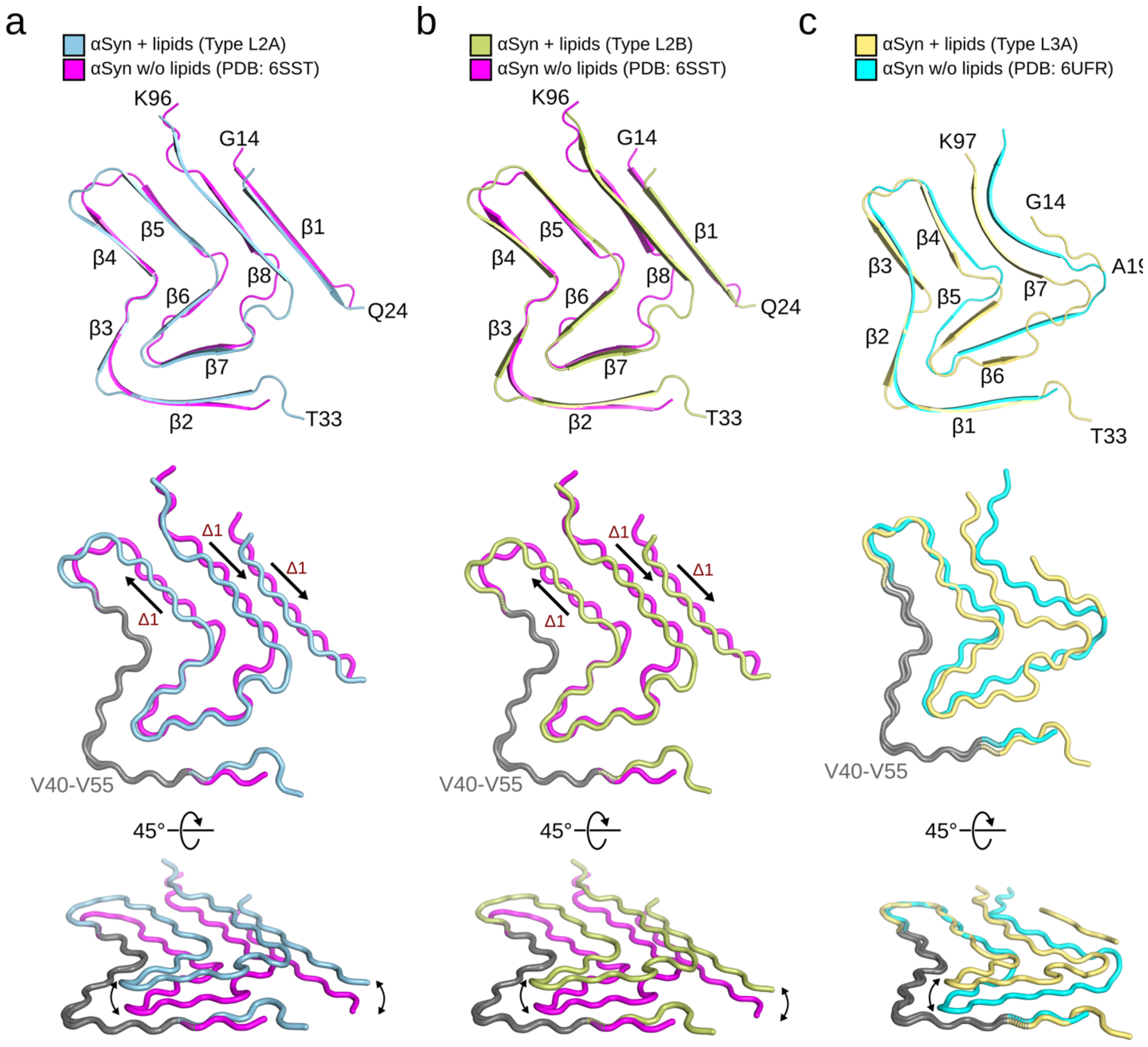

Supplementary Figure 6 | Comparison between *L2* and *L3* fibrils and known structures. Superposition of a single protein chain of (**a**) L2A αSyn onto in vitro aggregated wild type αSyn (PDB: 6SST [5]; Cα RMSD = 2.9 Å), (**b**) L2B αSyn onto in vitro aggregated wild type αSyn (PDB: 6SST [5]; Cα RMSD = 3.0 Å), and (**C**) L3A αSyn onto in vitro aggregated E46K αSyn (PDB: 6UFR [9]; Cα RMSD = 3.0 Å). Termini and β-strands are labeled. The middle panel visualizes the relative shift of β1, β5, and β8 introduced by the presence of lipids, after superimposing V40 to V55 (gray). The lower panel indicates the out-of-plane shift induced by the presence of lipids, as shown on a single chain.

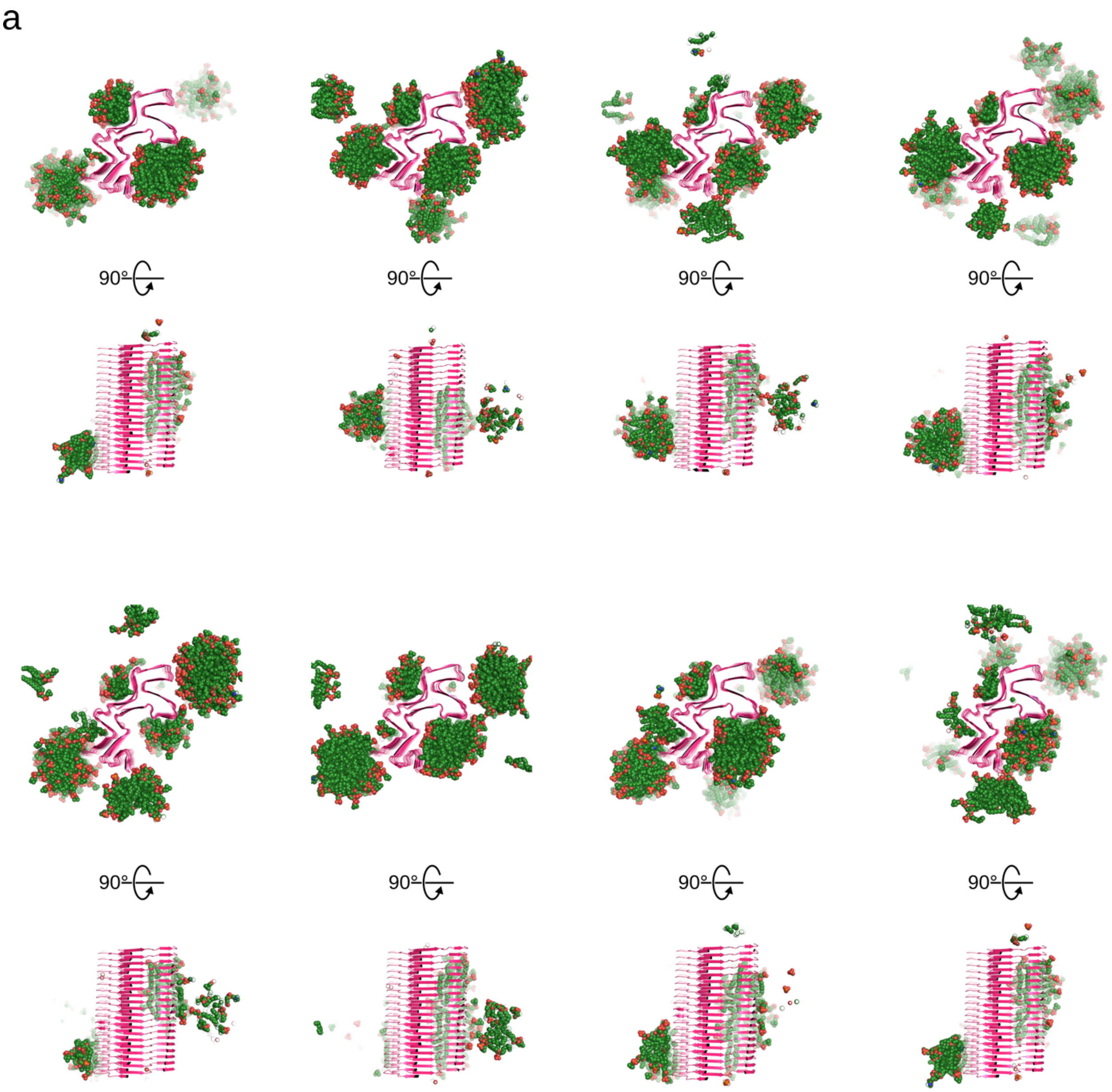

100 **Supplementary Figure 7 (consecutive)**

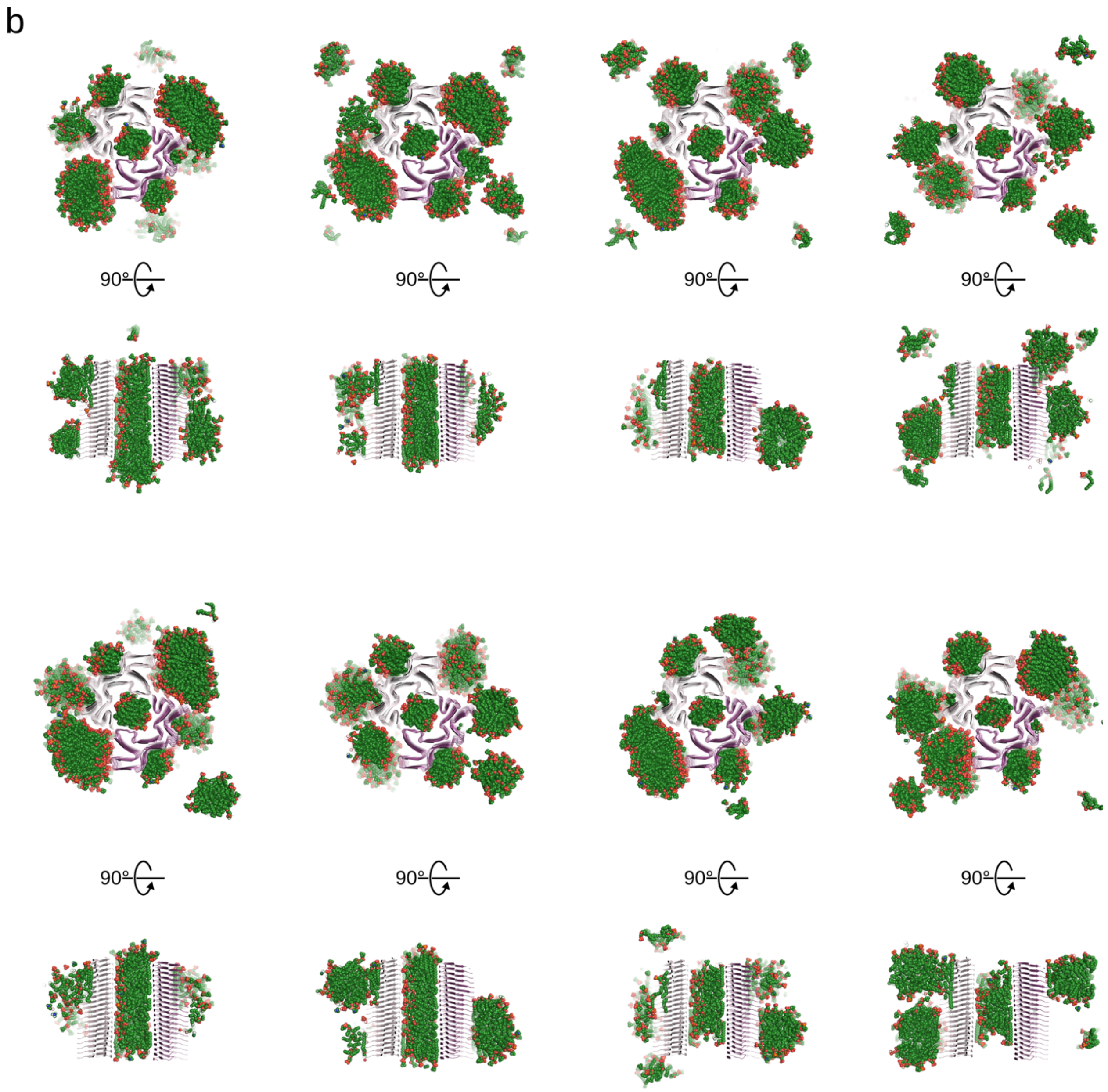

Supplementary Figure 7 (consecutive)

c

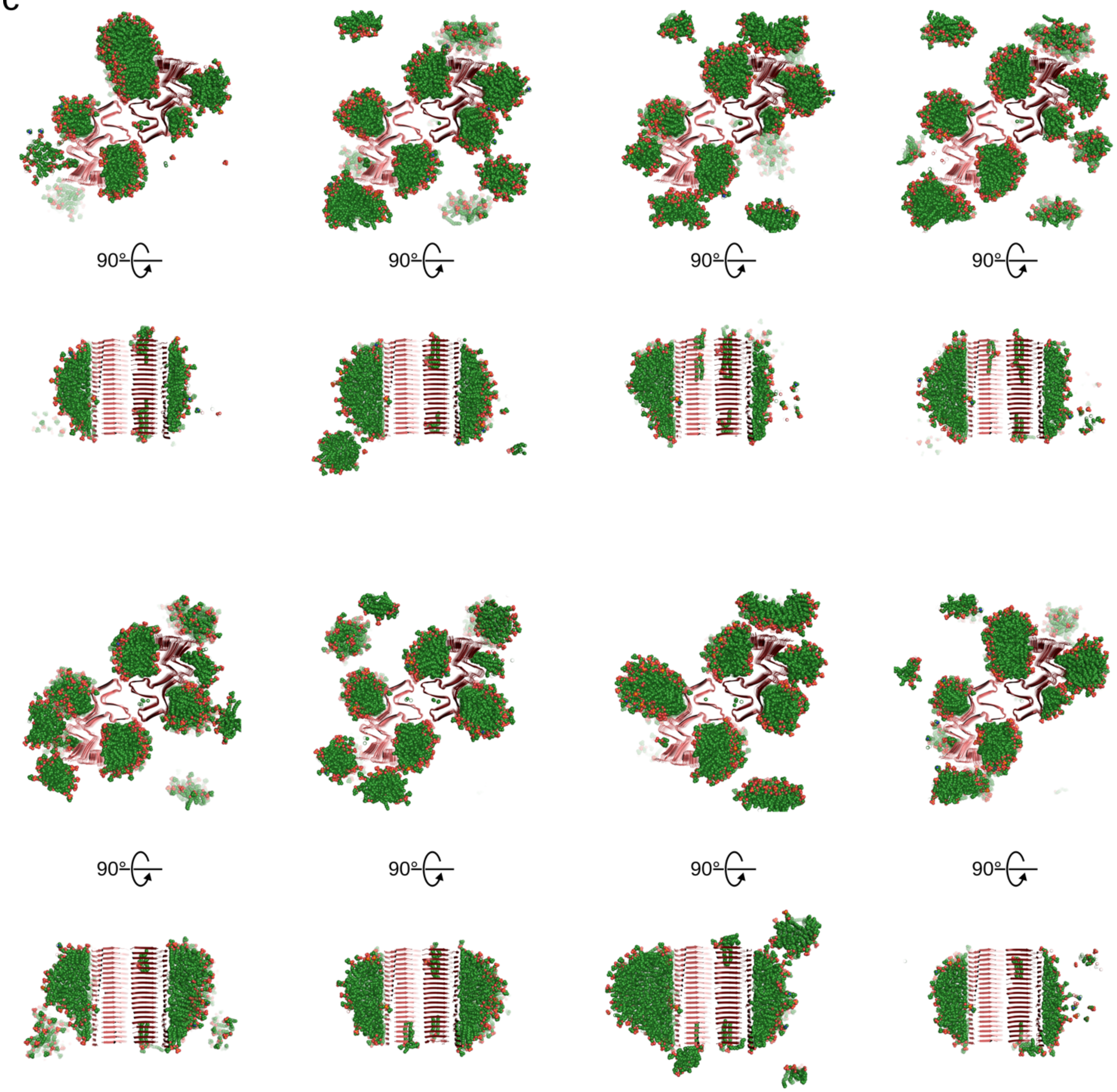

Supplementary Figure 7 (consecutive)

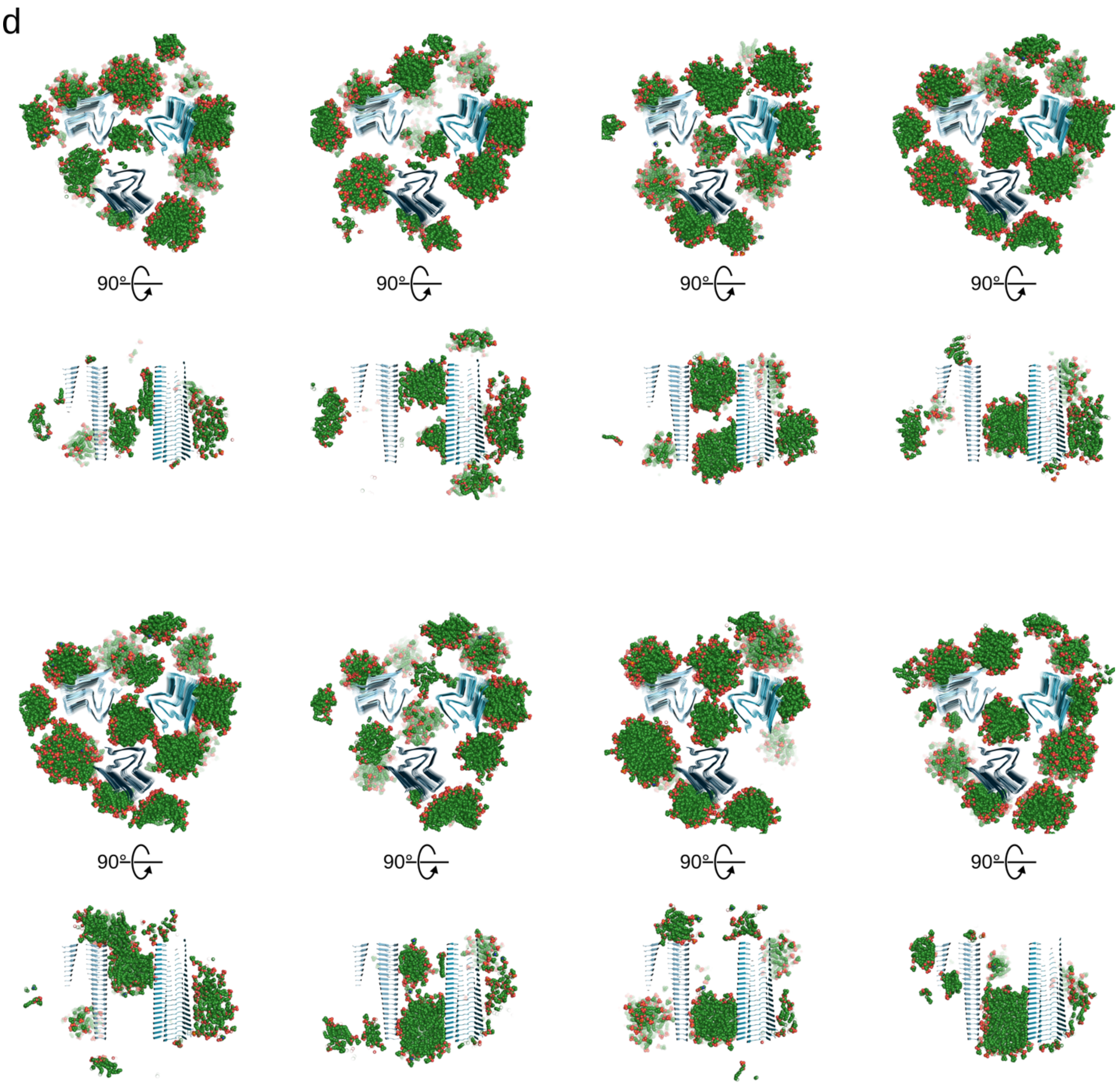

Supplementary Figure 7 (consecutive)

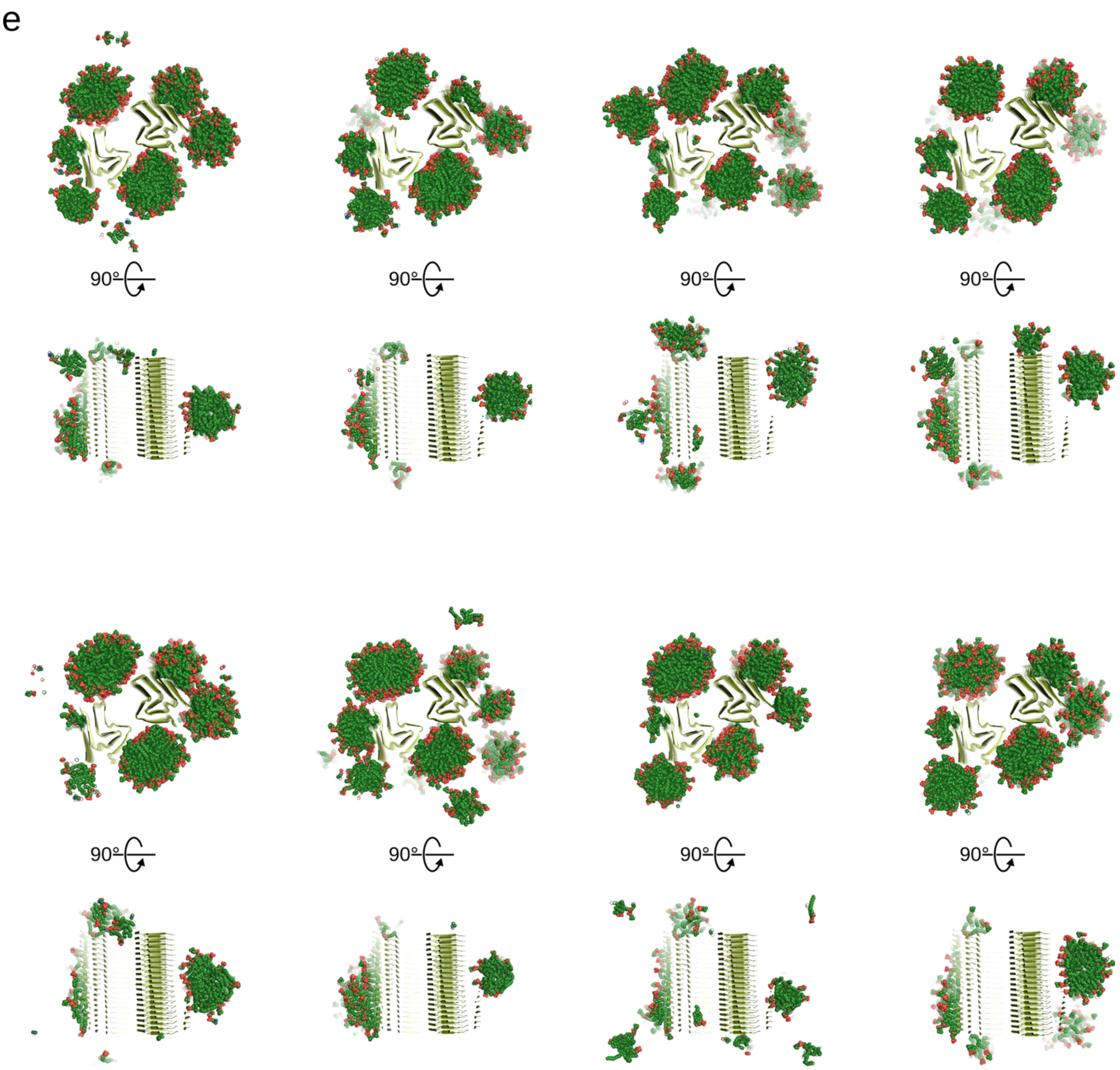

Supplementary Figure 7 (consecutive)

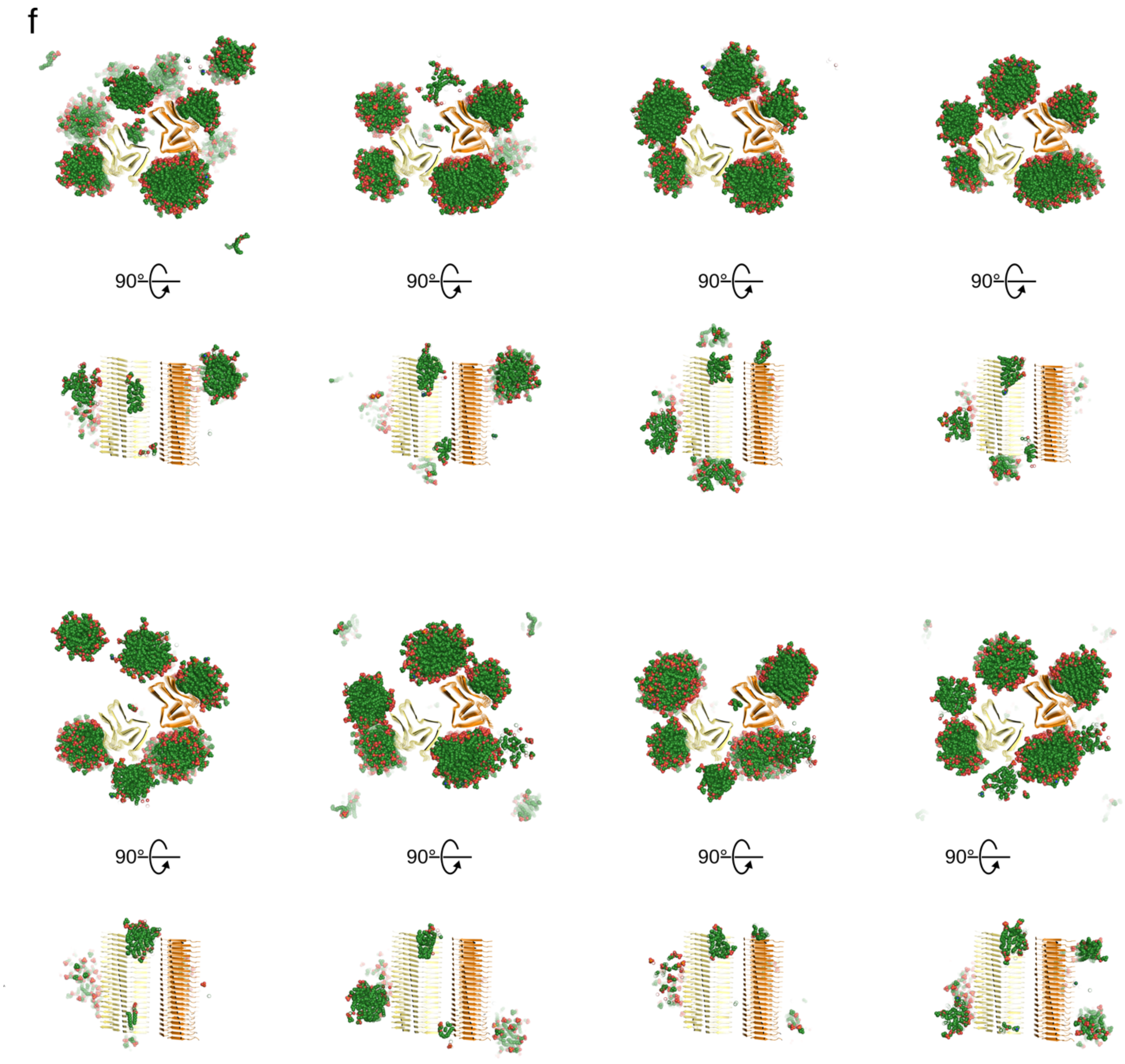

110 **Supplementary Figure 7 | Molecular dynamics simulations of the unbiased lipid diffusion.** Cross-section through the conformations after eight 1 μs MD simulations of unbiased lipid diffusion in the presence of the lipidic for L1A (**a**), L1B (**b**), L1C (**c**), L2A (**d**), L2B (**e**), or L3A (**f**) fibril, viewed from two perspectives. The fibril is shown as cartoon, the lipids as green spheres.

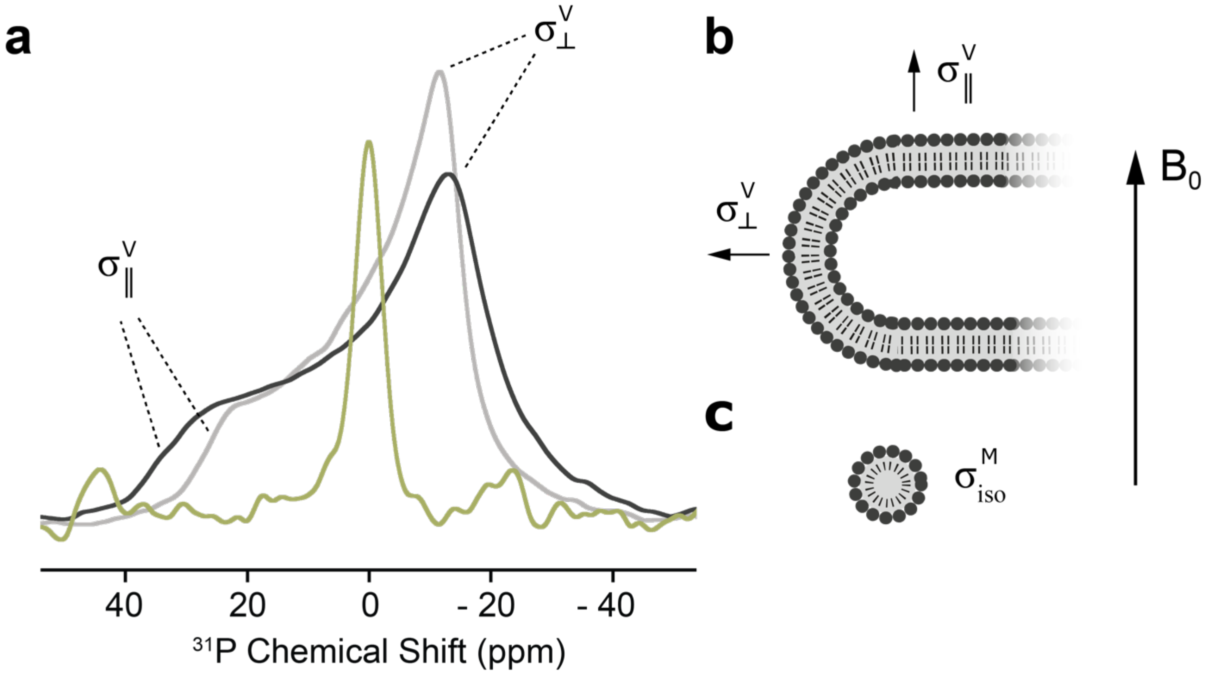

Supplementary Figure 8 | The conversion of the SUVs to small lipid aggregates during fibril preparation.

a, ^{1}H decoupled static ^{31}P ssNMR spectra of vesicles of POPA and POPC (1:1) at 280 K (black) and 310 K (grey) compared to the spectrum of lipidic αSyn fibrils at 280 K (green, same sample as dataset 2). Spectra of vesicles show a characteristic powder pattern due to chemical shift anisotropy (CSA) after uniaxial diffusion of the lipid molecule about its own long axis. Lateral diffusion of lipid molecules does not result in significant reorientation, consistent with lipid bilayer structures of low curvature (**b**). Lipids bound to αSyn fibrils show a single sharp line, indicating that CSA is averaged via isotropic reorientation of lipid headgroup moieties, consistent with the presence of high-curvature lipid aggregates, such as micelles (**c**). This behavior cannot be explained by a change of the lipid transition temperature and a resulting increase in mobility, since even at higher temperatures the vesicle spectra do not show a comparably sharp line.

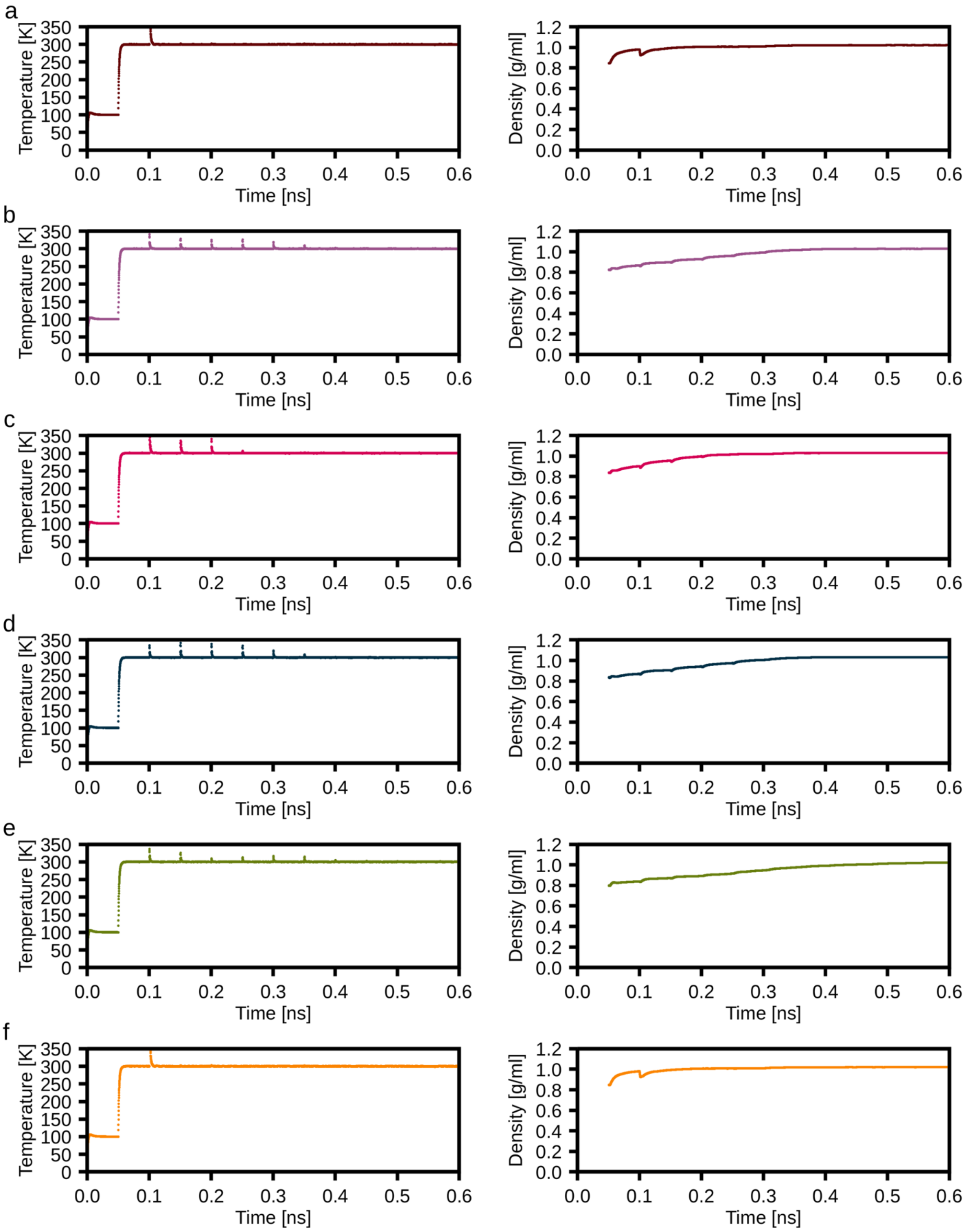
a
b
c
d
e
f
Temperature [K]
350
300
250
200
150
100
50
0
Density [g/ml]
1.2
1.0
0.8
0.6
0.4
0.2
0.0
0.0 0.1 0.2 0.3 0.4 0.5 0.6
Time [ns]

Supplementary Figure 9 | Thermalization and density adaptation data of molecular dynamics simulations.

Time series of the temperature (left panels) and density (right panels) over 0.6 ns of MD simulations for L1A (**a**), L1B (**b**), L1C (**c**), L2A (**d**), L2B (**e**), and L3A (**f**). The MD simulation procedure[10] started by heating the systems from 0 K to 100 K in a canonical (NVT) MD simulation of 50 ps length. Afterward, the temperature was raised from 100 K to 300 K during 50 ps of isobaric-isothermal (NPT) MD. Subsequently, the density was gently adjusted to 1 g/ml during 200 ps of NPT-MD. During the heating and density adaptation steps, positional restraints of 1 $kcal\cdot mol^{-1}\cdot Å^{-2}$ were applied to all backbone and lipidic phosphate atoms. These harmonic positional restraints were removed from the lipidic phosphate atoms by gradually decreasing the force constant from 1 to 0 $kcal\cdot mol^{-1}\cdot Å^{-2}$ in six NPT-MD runs of 50 ps length each. From here, we (re-)started eight independent NPT production simulations at 300 K and 1 bar for 1 µs each, in that new velocities were assigned from Maxwell-Boltzmann distribution. Importantly, we restrained the backbone to the initial atomic coordinates throughout production simulations while all other atoms were allowed to move freely.

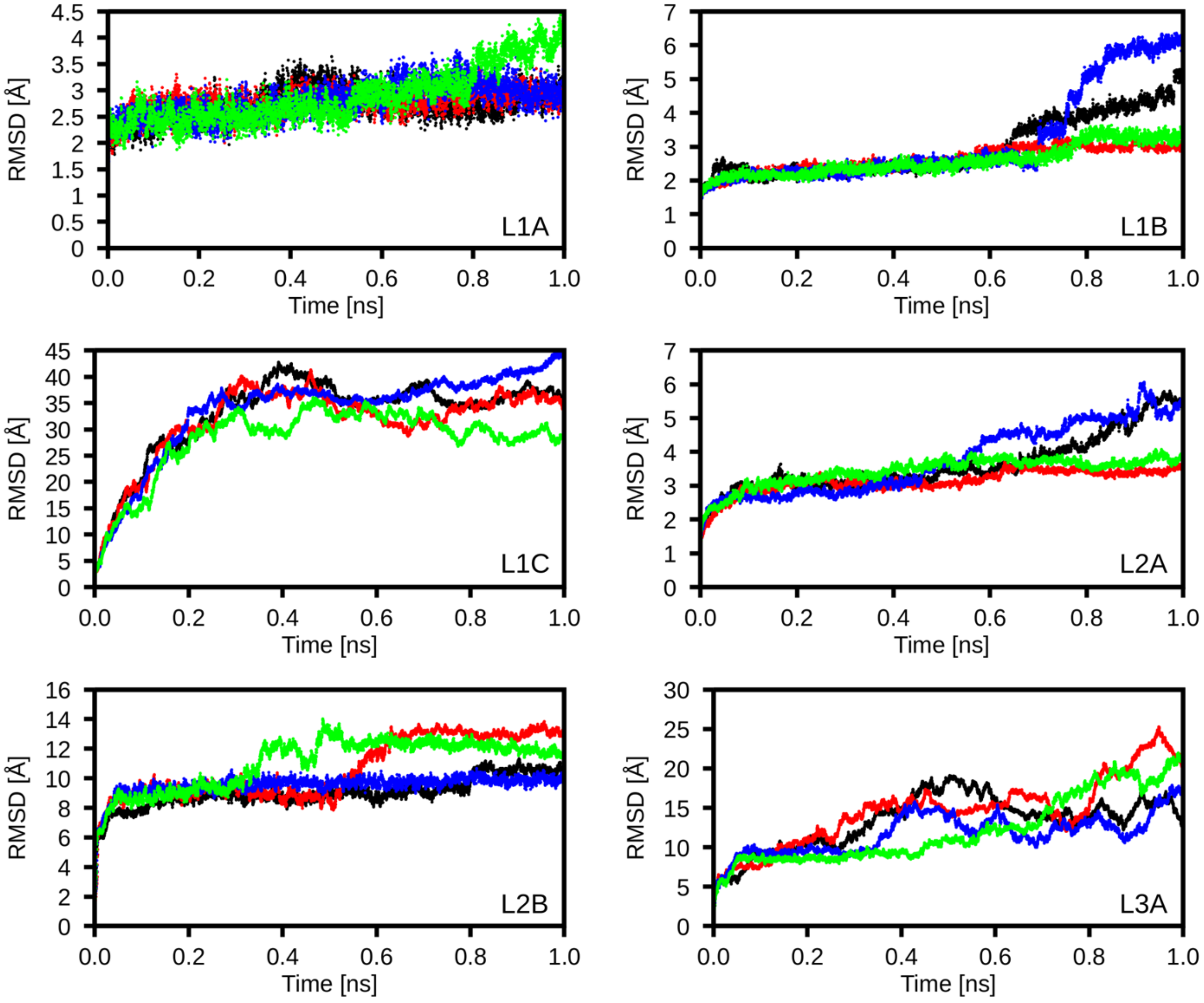

Supplementary Figure 10 | Structural analyses of fibrils during MD simulations without positional restraints and without lipids.

The plots show the backbone root mean square deviations (RMSD) throughout four replica (colored differently) MD simulations of 1 µs length relative to the starting structures. In contrast to the simulations used to calculate the distribution of phospholipids (**Figure 3**), here we performed simulations without any positional restraints and in the absence of phospholipids, leading to increased RMSD values, which indicates pronounced structural deviation from the starting structure. In the case of L2A, we only simulated one protofilament, since the three protofilaments were not in contact at all.

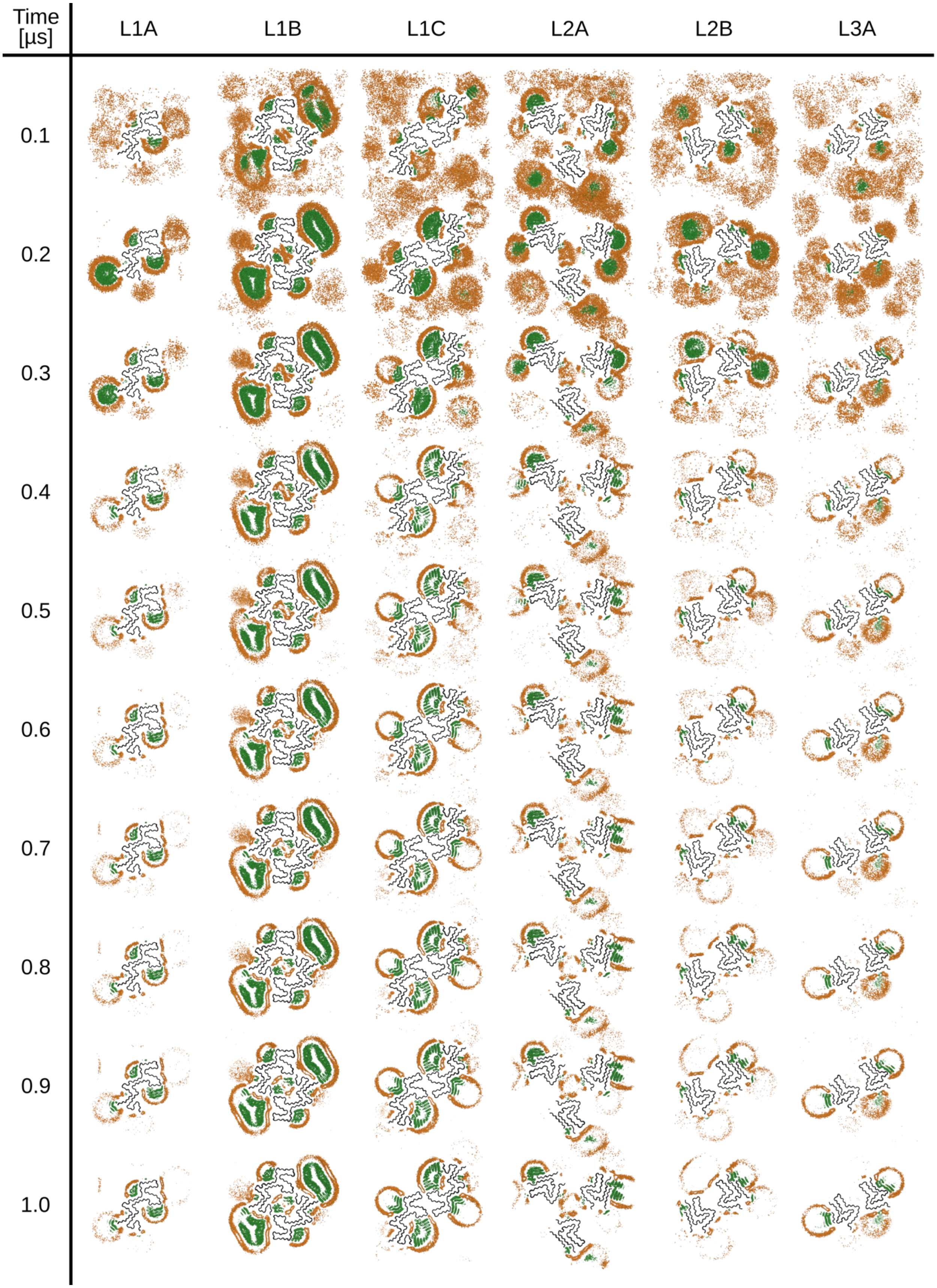
Time [µs]
L1A
L1B
L1C
L2A
L2B
L3A
0.1
0.2
0.3
0.4
0.5
0.6
0.7
0.8
0.9
1.0

Supplementary Figure 11 | Progression of lipid density grids during single replica simulations of all six fibrils.
Cross-section views (the central 15 Å of the fibrils) showing grids indicating the probability density of the lipid acyl chain (dark green) and lipidic phosphate atoms (orange) with increasing simulation time (in 0.1 µs intervals). The fibrils' backbones are shown as black ribbons.

Supplementary Table 1 | Cryo-EM structure determination statistics.

**Lipid-induced PM**	**L1A**	**L1B**	**L1C**	**L2A**	**L2B**	**L3A**
**Data collection**						
Microscope	Titan Krios G2	Titan Krios G2		Titan Krios G2		
Voltage [keV]	300	300		300		
Detector	K3	K3		K3		
Magnification	81,000	81,000		81,000		
Pixel size [Å]	1.05	1.05		1.05		
Defocus range [µm]	-0.5 to -2.0	-0.7 to -2.0		-0.5 to -2.0		
Exposure time [s/frame]	2.997	2.997		2.997		
Number of frames	40	50		40		
Total dose [e$^-$/Å^2]	42.72 (1.07 e$^-$/Å^2/frame)	50.83 (1.02 e$^-$/Å^2/frame)		42.72 (1.07 e$^-$/Å^2/frame)		
**Reconstruction**						
Micrographs	4,589	4,324		4,542		
Box width [pixels]	250	250		250		
Inter-box distance [pixels]	14	14		14		
Picked segments (no.)	585,342	504,236		1,223,706		
	L1A	L1B	L1C	L2A	L2B	L3A
PDB-ID	8ADU	8ADV	8ADW	8A4L	8ADS	8AEX
EMDB-ID	15370	15371	15372	15148	15369	15388
Final segments [no.]	13,641	19,108	25,817	46,003	20,388	46,882
Final resolution [Å] (FSC=0.143)	3.24	2.98	2.95	2.68	3.05	2.76
Applied map sharpening B-factor [Å^2]	-85.24	-83.67	-87.28	-98.95	-78.72	-85.99
Symmetry imposed	C1	C1	C2	C3	C1	C1
Helical rise [Å]	4.69	2.37	4.69	4.68	4.69	4.72
Helical twist [°]	-0.96	179.49	-0.72	-0.75	-0.82	-0.95

Supplementary Table 2 | Model building statistics.

**Lipid-induced PM**	**L1A**	**L1B**	**L1C**	**L2A**	**L2B**	**L3A**
**Initial model [PDB code]**	de novo	de novo	de novo	6SST	6SST	6UFR
**Model composition**						
Chains	5	10	10	15	10	10
Non-hydrogen atoms	3,460	6,920	6,920	7,755	5,170	4,665
Protein residues	495	990	990	1,125	750	680
**RMS deviations**						
Bond lengths [Å]	< 0.01	< 0.01	0.01	< 0.01	< 0.01	0.01
Bond angles [°]	0.82	0.64	1.5	0.65	0.42	1.16
**Validation**						
MolProbity score	2.39	2.37	2.95	1.53	1.32	2.49
Clashscore	20.22	16.36	8.01	10.11	5.84	12.49
**Ramachandran plot**						
Outliers [%]	0	0	0	0	0	0
Allowed [%]	11.34	9.28	7.73	0	0	8.46
Favored [%]	88.66	90.72	92.27	100	100	91.54

Supplementary Movie S1. Lipid binding to the *L1B* αSyn fibril.

The movie shows the first 100 ns of a representative trajectory of randomly placed phospholipids (1:1 mixture of POPC/POPA) binding to the *L1B* αSyn fibril. The lipids are shown as green-sphere model, and the αSyn fibril as cartoon, with both protofilaments colored differently.

Supplementary References

1. Meisl, G. et al. Molecular mechanisms of protein aggregation from global fitting of kinetic models. *Nat. Protoc.* **11**, 252-272 (2016).
2. Antonschmidt, L. et al. Insights into the molecular mechanism of amyloid filament formation: Segmental folding of alpha-synuclein on lipid membranes. *Sci. Adv.* **7**, eabg2174 (2021).
3. Zhao, K. et al. Parkinson's disease-related phosphorylation at Tyr39 rearranges alpha-synuclein amyloid fibril structure revealed by cryo-EM. *Proc. Natl. Acad. Sci. U. S. A.* **117**, 20305-20315 (2020).
4. Schweighauser, M. et al. Structures of alpha-synuclein filaments from multiple system atrophy. *Nature* **585**, 464-469 (2020).
5. Guerrero-Ferreira, R. et al. Two new polymorphic structures of human full-length alpha-synuclein fibrils solved by cryo-electron microscopy. *Elife* **8**, e48907 (2019).
6. Guerrero-Ferreira, R. et al. Cryo-EM structure of alpha-synuclein fibrils. *Elife* **7**(2018).
7. McGlinchey, R.P., Ni, X.D., Shadish, J.A., Jiang, J.S. & Lee, J.C. The N terminus of alpha-synuclein dictates fibril formation. *Proc. Natl. Acad. Sci. U. S. A.* **118**(2021).
8. Eisenberg, D., Schwarz, E., Komaromy, M. & Wall, R. Analysis of membrane and surface protein sequences with the hydrophobic moment plot. *J. Mol. Biol.* **179**, 125-142 (1984).
9. Boyer, D.R. et al. The alpha-synuclein hereditary mutation E46K unlocks a more stable, pathogenic fibril structure. *Proc. Natl. Acad. Sci. U. S. A.* **117**, 3592-3602 (2020).
10. Frieg, B. et al. Molecular mechanisms of glutamine synthetase mutations that lead to clinically relevant pathologies. *PLoS Comput. Biol.* **12**, e1004693 (2016).
