# Supplementary material for: The 3D structure of lipidic fibrils of α-synuclein
Source: Nat Commun. 2022 Nov 10;13:6810. doi: 10.1038/s41467-022-34552-7 (PMC9649780; doi:10.1038/s41467-022-34552-7)
Supplement: Supplementary file 3 — Description of Additional Supplementary Files [file 41467_2022_34552_MOESM3_ESM.docx]

**Description of Additional Supplementary Files**

File Name: Supplementary Movie 1

Description: Lipid binding to the L1B αSyn fibril. The movie shows the first 100 ns of a representative trajectory of randomly placed phospholipids (1:1 mixture of POPC/POPA) binding to the L1B αSyn fibril. The lipids are shown as green-sphere model, and the αSyn fibril as cartoon, with both protofilaments colored differently.
